# Benefits of Combined Surgical Techniques in Otoplasty

**DOI:** 10.5152/eurasianjmed.2022.22037

**Published:** 2022-10-01

**Authors:** Abdulkerim Olgun, Muhammet Dilber

**Affiliations:** 1 Department of Plastic Reconstructive and Aesthetic Surgery, Atatürk University Faculty of Medicine, Erzurum, Turkey; 2 Dilber ENT and Aesthetic Clinic, İstanbul, Turkey

**Keywords:** Otoplasty, prominent ear, conchamastoid suture

## Abstract

**Objective:**

Prominent ear deformity is one of the aesthetic problems that should be treated due to the psychosocial problems it causes. We aimed to present our results in which we used the combination of Mustarde suture to create an antihelix, conchal resection, and concomastoid suture combination to reduce conchal excess in the light of literature.

**Materials and Methods:**

A total of 28 cases in which the same otoplasty method was used in 2 separate centers between 2014 and 2020 were included in the study. Elliptic skin excision, antihelix reconstruction with Mustarde suture, and in cases with large conchal cartilage, cartilage excision and reconstruction with concomastoid suture were performed in all of our cases. After the skin was closed, a bandage was applied to the ear.

**Results:**

In the early postoperative period, hematoma developed in 4 cases. There was superficial erosion of the skin in 4 cases. In the late period, hypertrophic scar developed in both ears and resolved with medical treatment. There was minimal asymmetry in 4 cases, but intervention was not required in cases with asymmetry. Skin necrosis, infection, and visible cartilage irregularity on the anterior side were not observed in any case.

**Conclusion:**

The aim of otoplasty is to create symmetrical ears with smooth contours, so combined techniques should be used instead of a single technique. In otoplasty, we used Mustarde suture to create antihelix, conchal resection, and concomastoid suture combination to reduce cavum. Our results were satisfactory.

## Introduction

The prominent ear is not a disease, it is an aesthetic problem caused by congenital and familial reasons. The term prominent ear includes more than 1 anomaly. The causes of prominent ear deformity occur mainly due to 3 main reasons. These are underdevelopment of the antihelix, excessive development of the conchal cartilage, or greater than normal conchamastoid angle. The incidence of prominent ears is around 5% in the Caucasian race and is one of the most common congenital anomalies with autosomal dominant inheritance.^1,2^ The deformity frequently seen in the studies is the combination of underdevelopment of the antihelix and overdevelopment of the conchal cartilage. Adamson et al^3^ reported that conchal protrusion and antihelical deformity were together in 90% of the cases.

The auricle is a flexible structure consisting of the main skeleton formed by the skin, subcutaneous adipose tissue, and cartilage and has many anatomical regions such as concha, helix, antihelix, tragus, antitragus, and lobule. Due to the fact that the ears are located on the visible part of the face, especially school-age children are exposed to the annoying jokes of other children, and this can cause psychological problems. Due to the psychological problems it caused, it became necessary to apply the surgical treatment.^4,5^ Due to the fact that the ears can be covered with hair, there is no functional deficiency, and the socio-economic situation of the societies, some cases, although rare, apply to the doctor in advanced ages. 

A wide variety of prominent ear surgery techniques have been described in the literature. Although guide tables are recommended in prominent ear surgery, determining the most appropriate technique for patients is still a matter of debate.^6^ Currently, suturing and cartilage cutting techniques are the 2 main surgical approaches most frequently preferred by surgeons.^4^ Many surgeons try to create their own standard method using the method they find safe or a combination of various methods.^6-11^ The first reported surgical approach was by Dieffenbach in 1845.^12^ The aim of the surgery is to create a natural antihelix and conchal cartilage and to correct the concomastoid angle. 

Today, many prominent ear correction procedures have been reported.^4^ In our study, we used Mustard’s suture to create the antihelical fold, conchal cartilage resection to reduce the conchal cartilage, and concomastoid suture to correct the concomastoid angle. We aimed to present our experience and results in cases with prominent ear deformity corrected with this combined technique in the light of literature.

## Material and Methods

In this retrospective study, 28 patients who were admitted to our outpatient clinic with the complaint of prominent ear and were operated for this reason between August 2014 and October 2020 were included. The study was started after the approval was taken from the Clinical Research Ethics Committee.

Before surgery, we informed all the patients regarding the operation and took the signed-informed consent of them. All of the patients included in the study were from primary surgeries, and revision surgeries were not included in the study. Patients who could not be followed up and had missing information in their files were also excluded from the study. The surgical method for the patients included in the study was the cases in which the combined technique was applied, which we have mentioned in detail above. Preoperative routine head and neck examination and anatomical structures of the auricle were evaluated, and preoperative planning was made accordingly.

### Surgical Method

**Figure 1.**
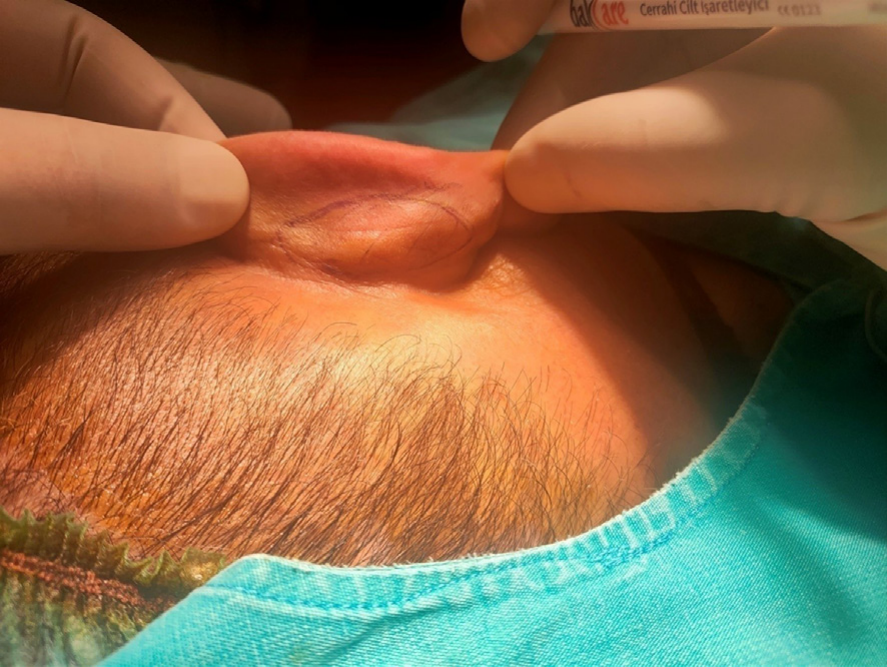
Skin area marked with methylene blue before posterior elliptical skin excision.

**Figure 2.**
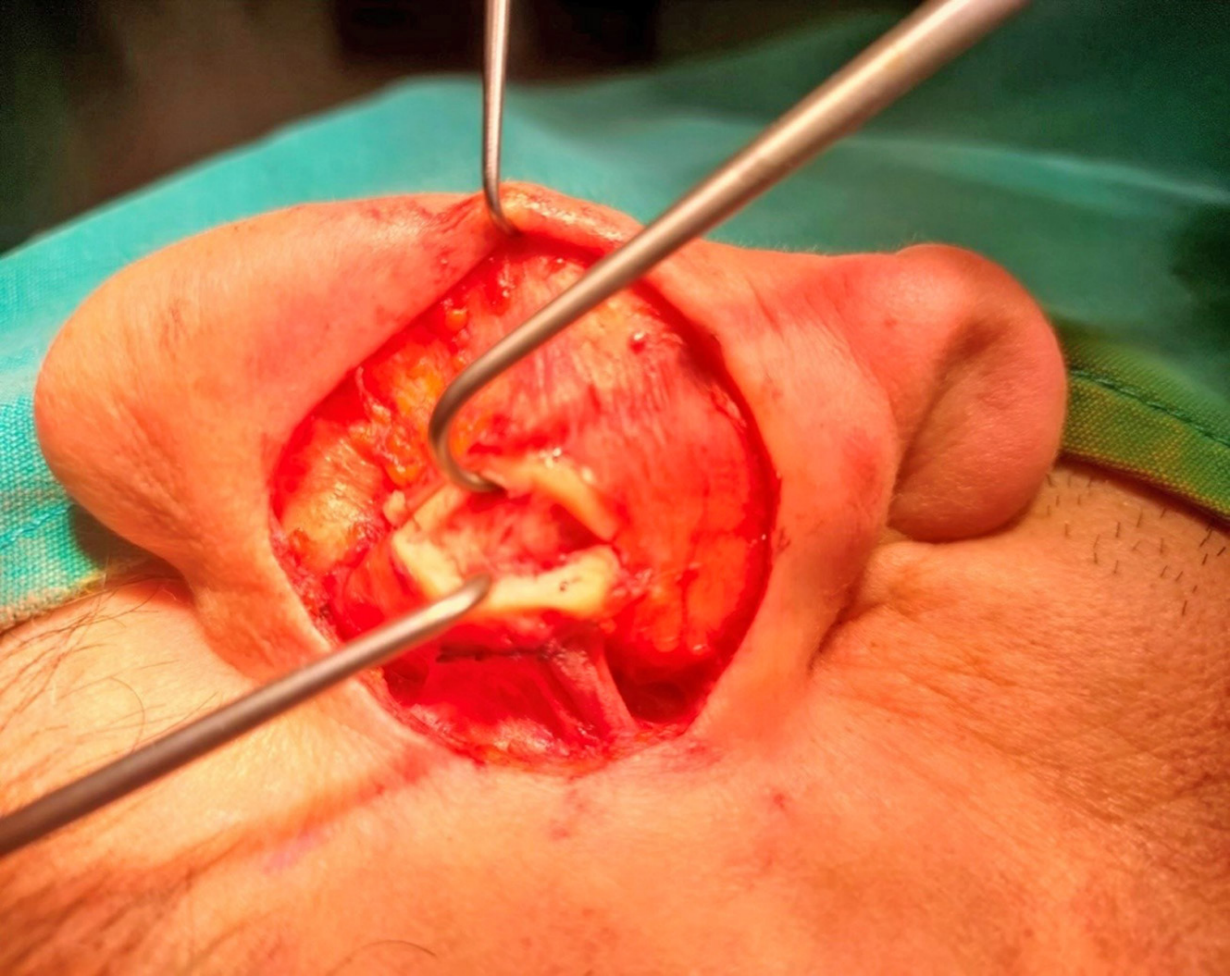
Excision of elliptical skin and conchal cartilage.

**Figure 3.**
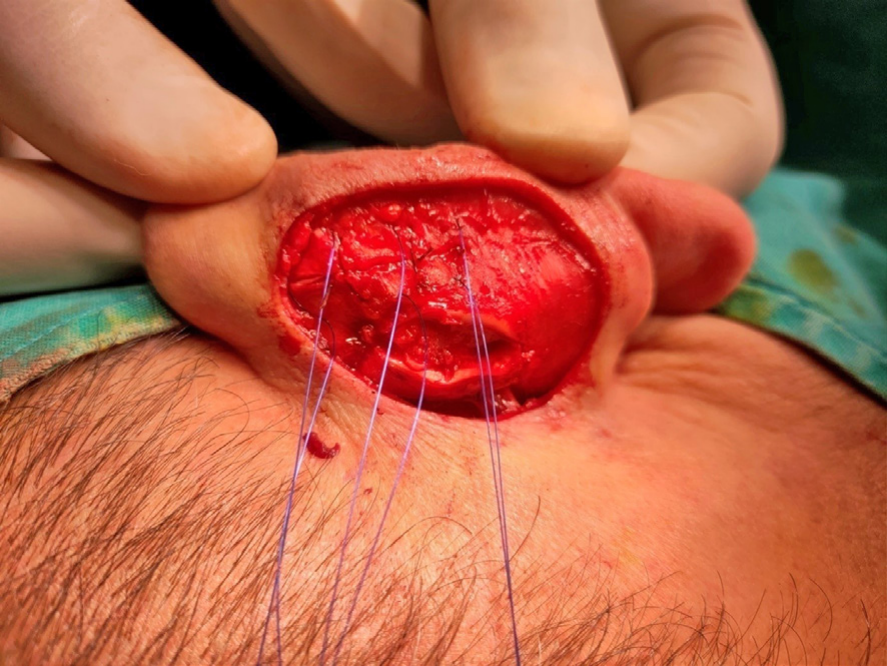
Horizontal mattress suturing with 5/0 polypropylene monofilament suture.

All our cases were operated under general anesthesia. After general anesthesia application, local anesthesia was applied to the auricle with an average of 10 mg of lidocaine and a local anesthetic substance containing 1/1000 adrenaline. After general anesthesia, the operation area was cleaned with antiseptics. Before local anesthetic application, the skin was marked with methylene blue before posterior elliptical skin excision (Figure 1). Local anesthesia was then administered with an average of 10 mg of lidocaine and a local anesthetic substance containing 1/1000 adrenaline. In all of our cases, elliptical skin excision was performed with a scalpel no. 15 from behind the ear, wide-ranging from the conchamastoid junction to the scaphal region. The incision borders on the skin were created 1 mm above the conchamastoid sulcus in the inferior and approximately 1 cm from the helix to the medial superiorly (Figure 2). Then, 0.5 cm incision was made from the posterosuperior of the auricle and the anterior of the auricle was reached in the subperichondral plane in the subperichondral plane. From here, the cartilage was made flexible with an injection needle along the antihelical fold line planned on the anterior surface of the auricle cartilage. Then, using 5/0 polypropylene monofilament suture, antihelical fold was created with horizontal mattress sutures technique (Figure 3). In the second stage, the required amount of excision was made from the most concave part of the conchal cartilage according to the depth in the conchal cartilage and the angulation caused by it (Figure 2). In the third stage, auricular angulation was corrected with concomastid sutures. After hemostasis with electrocautery, the skin was closed with 5/0 polypropylene monofilament suture. After the skin was closed, mupirocin 2% ointment-impregnated tampons were placed on the anterior surface of the auricula to support the newly formed antihelix and postauricular sulcus. In all cases, the dressings were removed on the second postoperative day and the operation area was evaluated. Compressed dressing was continued for 7 more days, then skin sutures were removed. After the pressure bandage was terminated, the patients were recommended to use tennis bands at night for 3 weeks. Antibiotherapy (oral ampicillin sulbactam or cefuroxime axetil) was administered to all of our patients for 1 week.

### Statistical Analysis

Statistical analyses were performed using Statistical Package for the Social Sciences software (IBM SPSS Corp.; Armonk, NY, USA) package program. Data were evaluated with descriptive statistics and independent samples *t*-test. A value of *P* < .05 was considered significant.

## Results

In the study, otoplasty was performed on 54 ears of 28 patients. Eight of the cases were male and 20 were female. Except for 4 cases, all cases were older than 18 years of age. The mean age was 22.6 years. Twenty-six cases were operated bilaterally and 2 cases unilaterally (Table 1). In all ears, horizontal mattress sutures, conchal cartilage excision, and conchamastoid sutures were used together. All cases were operated under general anesthesia. In the early postoperative period, hematoma developed in 4 cases, only 1 had drainage, and the others healed without drainage. There was mild skin erosion due to excessive pressure in 4 cases (healed without intervention). No infection was observed in any of our cases. Two of our patients had hypertrophic scarring in both ears and were healed with intralesional steroid injection (40 mg triamcinolone acetonide). In our other 4 cases, there was minimal asymmetry between the 2 ears, and in cases with asymmetry, no intervention was required, as the patients described the result as “acceptable.” In 2 of our cases, partial asymmetry due to suture rupture occurred in 2 ears and the problem was resolved with limited revision surgery. Skin necrosis, infection, and anterior cartilage irregularity were not observed in any of the cases. None of the patients in our study had complications such as foreign body reaction due to the suture material encountered in permanent sutures during the follow-up period (Table 2)

## Discussion

Although many surgical techniques have been described for prominent ear repair, no single technique is sufficient for the repair of all prominent ear cases.^13^ Since the anatomical features and severity of prominent ear deformities may differ in each patient, sometimes combined approaches are needed instead of a standard surgical technique.^14^ In prominent ear repair, it is aimed to keep the angle between the mastoid plane and the helix below 40° to bring the distance between the helix and the skull up to 1.5-2 cm and to provide symmetry between the 2 ears. Providing symmetry in ear deformities is the most basic goal of the surgery and the most important step that directly affects the result.^13^

In otoplasty cases, underdevelopment of the antihelix, hypertrophy of the concha, wider than normal conchamastoid angle, and lobule hypertrophy are common anomalies. In many studies, mostly antihelix-related deformities have been seen and techniques related to this deformity have been defined mostly.^1,15^ To create a new antihelix, 3 main methods that are frequently preferred are suturing, anterior notching, and incision techniques.^16^ The most well-known suture technique is Mustarde’s “mattress suture” technique.^1,17^ The Mustardé otoplasty demonstrated high efficacy in the correction of the prominent ear, with low reoperation rates and high patient and surgeon satisfaction.^18^ Chong Chet^19^ and Stenström^20^ suggest anterior notching. In this method, the basic mechanism is to notch one side of the helical cartilages to bend them toward the opposite side. In other studies, the techniques defined to create the antihelical fold were classified under 3 main headings: suturing and repair techniques, cartilage incision, and rasping procedures and their combination.^21,22^ Especially in patients with thick auricular cartilage, recurrence of the deformity can be observed in the long term after surgery, depending on the cartilage memory. The antihelical fold created using full-thickness cartilage removal and suturing sometimes has very sharp and unnatural lines. This situation reduces the postoperative satisfaction of the patients. In our study, we made an intervention to break the resistance of the cartilage before applying the mattress suture technique. On the anterior surface of the ear, the area corresponding to the antihelical fold was weakened in the suppericondral plane with the injection needle. Thus, a more effective and permanent antihelical fold was created. With this method, it has been observed that cartilage resistance is broken and recurrence is reduced, especially in ears with thick cartilage. In a study of 562 cases, complications such as recurrence of deformity, bad shape, and infection were detected as high as 20% in cases performed by new surgeons, while this rate was found to be around 9% in experienced surgeons. In the study, it was shown that the reason for the unsatisfactory results was the design of the operation in 73.4% of the cases and the unsuitability of the surgical technique in the second place in 26.6% of the cases. The most common complication was found to be patient dissatisfaction (8%) due to residual deformity.^23^ Complications occurring within the first 14 days after otoplasty are considered early complications. The most common early complications are hematoma and infection.^24^ In the study by Cıncık et al.^1^ the rate of major complications (necrosis, infection, and hematoma) was 3 (9.3%) in a series of 32 ears. This complication was hematoma in 1 ear and was treated with needle aspiration without causing any deformity. In another case, unilateral revision otoplasty was performed due to insufficient cosmetic results due to asymmetry, and in another case, due to exposure of the sutures by irritating the skin in the fourth week postoperatively. Minor complications (15.5%) developed in a total of 5 ears. These were minimal anterior protrusion of the lobule anteriorly in 1 case and bilaterally in 1 case. In the other 3 cases, the sutures were so superficial that they could be seen under the skin.^1^

The main goal of many surgical procedures based on this information is to intervene in the deformed area. In our study, we presented a holistic approach to complex prominent ear deformities. While creating the antihelical fold, we minimized the risk of recurrence by weakening the cartilage together with the mattress suture and excision from the conchal cartilage. 

The limitation of our study was not able to follow up our patients for a long time. And also we could not receive their magnetic resonance imaging result. We think that it needs further studies.

As a result, various surgical techniques have been defined for prominent ear deformity repair depending on the type of deformity, accompanying additional problems, and severity. Otoplasty is an operation that is very satisfying for the patient and the doctor. The aim is to create symmetrical ears with smooth contours, so combined techniques should be used instead of a single technique. In otoplasty, we used Mustarde suture to create antihelix, conchal resection, and concomastoid suture combination to reduce cavum. Our results were satisfactory.

## Figures and Tables

**Table 1.** Demographic and Clinical Characteristics of the Cases

**Demographic and Clinical Characteristics**	
**Average age and range (years)**	22.6 (18-27)
**Gender**	
**Male**	8 (28.6%)
**Female**	20 (71.4%)
**Otoplasty surgery**	
**One-sided**	2 (8.2%)
**Double-sided**	26 (92.8%)

**Table 2.** Complication of Otoplasty in Early and Late Period

**Complications**	**Early Stage**	**Late Stage**
**Hematoma**	7.4 (4)	0.0
**Erosion on the skin**	7.4 (4)	0.0
**Wound site infection**	0.0	0.0
**Kelloid development**	0.0	7.4 (4)
**Suture opening**	0.0	3.7 (2)
**Asymmetry**	0.0	7.4 (4)
**Recurrence**	0.0	3.7 (2)
